# P-222. Emergence and dissemination of *Clostridioides difficile* isolates with reduced fidaxomicin susceptibility in an acute care hospital

**DOI:** 10.1093/ofid/ofae631.426

**Published:** 2025-01-29

**Authors:** Sarah N Redmond, Jennifer Cadnum, Annette Jencson, Hosoon Choi, Munok Hwang, Piyali Chatterjee, Chetan Jinadatha, Curtis Donskey

**Affiliations:** University Hospitals Cleveland Medical Center, Cleveland, Ohio; Northeast Ohio VA Medical Center, Cleveland, Ohio; Cleveland VA Hospital, Cleveland, Ohio; Central Texas Veterans Health Care System, Temple, Texas; Central Texas Veterans Health Care System, Temple, Texas; Central Texas Veterans Health Care System, Temple, Texas; Central Texas Veterans Health Care System, Temple, Texas; Cleveland VA Hospital, Cleveland, Ohio

## Abstract

**Background:**

Despite increasing use of fidaxomicin for *Clostridioides difficile* infection (CDI), isolates with reduced susceptibility (minimum-inhibitory concentration [MIC] >2 ug/mL) have been rare in surveillance studies. However, there has been recent reports of clinical isolates with reduced susceptibility associated with mutations in the *RpoB* and *RpoC* genes coding for the RNA polymerase target of fidaxomicin. The clinical implications of reduced susceptibility are uncertain because fidaxomicin achieves high concentrations in the intestinal tract.

Whole genome sequencing analysis demonstrating that isolates from 3 patients (patients 1, 3, and 4) infected with ribotype 097 strains (sequence type [ST] 21) are genetically related (<2 single nucleotide polymorphism differences) suggesting patient-to-patient transmission

**Figure:**
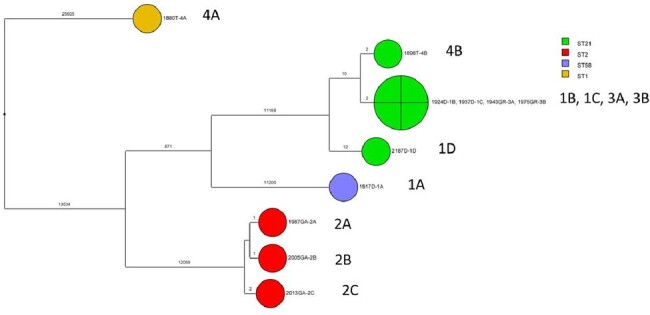

ST21 = Ribotype 097; ST2 = Ribotype 014-020

**Methods:**

During a 3-year period, patients treated for CDI in an acute care hospital were assessed for recurrence and fidaxomicin treatment failure, defined as persistent symptoms and culture positivity after >10 days of fidaxomicin treatment without an alternative explanation. Serial stool samples from these patients were cultured for *C. difficile* confirmed by MALDI-TOF. Susceptibility testing was performed by agar dilution. Whole genome sequencing (WGS) was performed to determine relatedness of the isolates (genetically related: <2 single nucleotide polymorphism differences) and to identify mutations associated with reduced fidaxomicin susceptibility.

**Results:**

Among 115 patients treated with fidaxomicin, there were 20 (17%) recurrences and 3 (2.6%) treatment failures. In 2 of the patients with treatment failure, initial *C. difficile* isolates were susceptible to fidaxomicin but genetically related isolates with reduced susceptibility (MIC >16ug/mL) associated with distinct mutations in *rpoB* (V1143G) and *rpoC* (R89G) emerged. The patient with the *rpoC* mutation had recurrent hospital admissions and maintained carriage of the isolate with reduced susceptibility over a 3-year period. Three additional patients were infected with *C. difficile* isolates with fidaxomicin MICs >8 ug/mL, including 2 patients with sequence type 21 (ribotype 097) isolates genetically related to the treatment failure isolate with the *rpoC* mutation (Figure).

**Conclusion:**

Emergence of *C. difficile* isolates with reduced fidaxomicin susceptibility may play a role in some cases of fidaxomicin treatment failure. Our findings suggest that patient-to-patient transmission could lead to dissemination of isolates with reduced fidaxomicin susceptibility.

**Disclosures:**

**Curtis Donskey, MD**, Clorox: Grant/Research Support|Pfizer: Grant/Research Support

